# Expression of RSK4, CD44 and MMP-9 is upregulated and positively correlated in metastatic ccRCC

**DOI:** 10.1186/s13000-020-00948-6

**Published:** 2020-03-24

**Authors:** Jing Ma, Mingyang Li, Jia Chai, Kaijing Wang, Peifeng Li, Yixiong Liu, Danhui Zhao, Junpeng Xu, Kangjie Yu, Qingguo Yan, Shuangping Guo, Zhe Wang, Linni Fan

**Affiliations:** 1 State Key Laboratory of Cancer Biology, Department of Pathology, Xijing Hospital and School of Basic Medicine, Fourth Military Medical University, Changle West Road #169, Xi’an, 710032 Shaan Xi Province China; 2 Department of Pathology, The 960th Hospital of PLA, Jinan, Shandong China

**Keywords:** Primary ccRCC, Metastatic ccRCC, RSK4, CD44, MMP-9, Prognosis

## Abstract

**Background:**

To investigate the expression and function of RSK4, MMP-9 and CD44 in primary clear cell renal cell carcinoma (primary ccRCC) and metastatic clear cell renal cell carcinoma (metastatic ccRCC), as well as the correlation with clinicopathological features of patients.

**Method:**

The expression levels of RSK4, CD44 and MMP-9 in 52 primary ccRCC samples and 48 metastatic ccRCC samples were detected by immunohistochemistry, and the relationship between RSK4, CD44 and MMP-9 expression and clinicopathological features as well as prognosis of metastatic ccRCC patients was statistically analysed. Ectopic RSK4 expression in ccRCC cell lines was performed to determine its effect on cell cycle regulation, tumour invasiveness, and metastatic capability.

**Results:**

The positive rates of RSK4, MMP-9 and CD44 expression in metastatic ccRCC tissues were 75, 68.75 and 91.7%, respectively, while the rates in primary ccRCC tissues were 44.2, 34.6 and 69.2%, respectively. Thus, the positive rates in metastatic ccRCC were higher than those in primary ccRCC (*P*_RSK4_ = 0. 002; *P*_MMP-9_ = 0. 002; *P*_CD44_ = 0. 001). However, the expression of RSK4, CD44 and MMP-9 was unrelated to age, gender, or metastatic sites (*P* > 0.05) but was related to WHO/ISUP nucleolar grade (*P*_RSK4_ = 0.019; *P*_CD44_ = 0.026; *P*_MMP-9_ = 0.049). In metastatic ccRCC, expression among the three proteins showed a positive correlation (*P* = 0.008). Moreover, expression between RSK4 and CD44 (*P* = 0.019) and MMP-9 and CD44 (*P* = 0.05) also showed positive correlations, whereas RSK4 and MMP-9 showed no significant correlation (*P* = 1.00). Molecular studies showed that overexpression of RSK4 could enhance the invasive and migratory abilities of ccRCC cell lines through the regulation of CD44 and MMP-9 expression and vice versa.

**Conclusions:**

The overexpression of RSK4, MMP-9 and CD44 is associated with the invasion and metastasis of ccRCC, indicating that they could be potential prognostic factors and serve as new potential therapeutic targets for ccRCC.

## Backgroud

Renal cell carcinoma (RCC) is the most common malignant tumor type of all genitourinary cancers, and morbidity increases rapidly [1]. Clinically, RCC can remain occult throughout the disease course for the majority of cases. During the early stage of RCC, no overt symptoms can be observed, and because of the lack of a specific standard of diagnosis, approximately twenty to 30 % of patients present with metastasis when initially diagnosed. RCC has a well-described propensity for systemic metastasis, with migration to the skeletal system, respiratory system and central nervous system. RCC is resistant to radiation and chemotherapy, and many patients who undergo curative surgical resection experience recurrence during subsequent follow-up, which causes great difficulties for the diagnosis and treatment of RCC [2]. Up to now, two major types of factors have been found to be involved in RCC metastasis: one is tumour angiogenesis factors that can promote metastasis, such as MMPs and CD44, and the other is tumour suppressors, such as VHL and PTEN. However, the exact mechanisms need to be further studied. Therefore, it is essential to explore new specific and effective factors for prognosis prediction as well as the therapeutic targets of metastatic RCC.

Ribosomal S6 protein kinase 4 (RSK4), belonging to the RSK family, was first identified as an X-linked gene in patients with mental retardation and plays a major role in cell growth and proliferation; however, its functions remain largely unknown [3]. There are only a few studies on the distribution of RSK4 mRNA in human normal tissues. In some studies, RSK4 has been considered a potential tumour suppressing gene, and it has been reported that RSK4 expression is reduced in some tumours, while RSK4 overexpression could inhibit the invasion and metastasis of tumour cells [4]. In our previous study, we used multiple human normal and tumour organ tissue arrays (TMA) to investigate the expression of RSK4 in different tissues and found that RSK4 was also expressed in normal human tissues. Strong positivity was observed in pancreatic ductal epithelial cells, salivary epithelial cells, sweat gland epithelial cells, and in B lymphocytes found in tonsil germinal centres. The expression of RSK4 in renal tubular epithelial cells, hepatocytes, cardiomyocytes, and endometrial epithelial cells was weak. In clear cell RCC, uterus clear cell carcinoma, ovarian serous papillary cystadenocarcinoma, and gastric adenocarcinoma demonstrated strong positivity for RSK4, whereas some tumours, such as breast cancer and hepatocellular carcinoma, manifested weak positivity. Overall, the expression of RSK4 in RCC was higher than that in normal kidney tissue, and overexpression of RSK4 in RCC was associated with a high risk of invasion and metastasis [5], suggesting that RSK4 may play a crucial role in tumour progression of RCC.

According to previous reports, the expression levels of MMP-9 and CD44 are also high in RCC. Previous studies have shown that with the stimulation of the Ras-MEK-ERK signalling pathway, MMP-9 can bind with CD44-ICD to take on further regulation [6]. Whether RSK4 can mediate the invasion and metastasis of renal cell carcinoma by CD44 and MMP-9 is a new direction proposed by this study.

We used tissue slides to study the expression of the RSK4, CD44 and MMP-9 proteins in human pRCC and mRCC tumour tissues by immunohistochemistry and found that the RSK4, CD44 and MMP-9 proteins were weakly expressed in pRCC and much more strongly expressed in mRCC tissues. This indicates that RSK4, CD44 and MMP-9 may have an oncogenic role in the development of RCC. To test this hypothesis, we evaluated the expression of RSK4, CD44 and MMP-9 in mRCC tissues, analysed the relationship between RSK4, CD44 and MMP-9 expression and the clinicopathological features of mRCC patients, and explored the potential molecular mechanisms of the RSK4, CD44 and MMP-9 pathways in RCC cell lines.

## Materials and methods

### Clinical data

A total of 100 consecutive patients with primary ccRCC between 2007 and 2016 were identified from the pathology archives of Xijing Hospital, and 52 pRCC and 48 mRCC samples were included in the study. The pRCC patients consisted of 45 males and 7 females with a mean age of 54.2 years (range: 19–80 years). Based on WHO/ISUP nucleolar grade, 41 cases were categorised as low grade (grade 1–2), and 11 cases were classified as high grade (grade 3–4). The mRCC patients consisted of 41 males and 7 females with a mean age of 58.4 years (range: 28–80 years). Based on WHO/ISUP nucleolar grade, 33 cases were categorised as low grade (grade 1–2), and 15 cases were classified as high grade (grade 3–4). The metastatic sites included bones (23), soft tissues (10), lungs (6), brains (5), paranephros (1) and gall bladder (1). The median follow-up time was 36 months (range: 6–99 months). All slides were re-examined by two pathologists to ensure correct diagnosis. The specimen collection and study procedures were approved by the Ethics Committee of Xijing Hospital.

### Immunohistochemistry

Paraffin-embedded sections and slides of 4 mm thickness were deparaffinised and treated with 3% H_2_O_2_ to block endogenous peroxidase activity. Proteolytic antigen retrieval was performed in pepsin for 30 min at 37 °C, followed by incubation in 10% bovine serum albumin (HyClone, USA) in PBS at room temperature for 10 min to block the non-specific antibody-binding sites. The sections were then incubated overnight at 4 °C with mouse monoclonal antibody against the human RSK4 protein (1:50 dilution; Sigma, USA), human CD44 protein (1:150 dilution; Sigma, USA), human MMP-9 protein (1:300 dilution; Sigma, USA) and humanβ-catenin protein (1:100 dilution; Sigma, USA). Later, a standard rapid EnVision technique (Dako, Denmark) was used to detect the protein conjugates and develop the colour. Finally, the sections were visualised after counterstaining with haematoxylin. Serial sections of RCC were run in parallel with the primary antibody replaced by PBS and rabbit IgG1 (Santa Cruz Biotechnology) as blank and negative controls.

### Evaluation of immunohistochemical staining

The sections were imaged under an optical microscope (BX51, Olympus, Tokyo, Japan), and the images were captured using DP2-BSW software (Olympus). Immunohistochemical staining was evaluated simultaneously by three observers who had no knowledge of the clinicopathological features of the patients. The H score system was used to score the distribution of positive cells as follows: the distribution (the percentage of positive cells) was recorded as A = 0–100, and the staining intensity was assessed as B, whereby none (not stained) = 0, light (light brown) = 1, mild (brown) = 2, and strong (dark brown) = 3. The scores for distribution and intensity were added and graded as follows: H score = 1 × a1 + 2 × a2 + 3 × a3, 0 ≤ H ≤ 300.

### Cell lines, plasmids, and transfection

The human RCC cell line ACHN was obtained from the Experimental Animal-raise Centre (Air Force Military Medical University, Xi’an, China) and cultured in RPMI 1640 (HyClone, Thermo, USA) with 10% foetal bovine serum (FBS; Gibco, Carlsbad, CA, USA) and 5% CO_2_ at 37 °C. The pcDNA3.1/Neo-RSK4 plasmid was used to stably transfect the RCC cell line to overexpress human RSK4. Transfection was performed using Lipofectamine 2000 (Invitrogen, Carlsbad, CA, USA) according to the manufacturer’s instructions. Briefly, cells were cultured in six-well plates to 90% confluence. Plasmid DNA (4.0 mg per well) was mixed with 10 ml Lipofectamine 2000 to transfect the RCC cells. After 48 h, cells were trypsinised and replated onto 10-cm culture dishes in the presence of 300 mg/ml G418 (Gibco). Single-cell clones were isolated for clonal expansion. Each cell clone was screened using reverse transcription-PCR and western blot analysis to determine RSK4 expression at both the mRNA and protein levels.

### Lentivirus-based shRNA transduction

RSK4 shRNA was provided by Genechem Co., Ltd. (Shanghai, China). Lentivirus-based transduction was carried out using a packaging cell line, HEK293T, for co-transfection of psPAX2 and pMD2.G (kindly donated by Professor Jian Zhang, Department of Biochemistry and Molecular Biology, Air Force Military Medical University, Xi’an, China). After two consecutive viral infections, transfected cells were selected using puromycin (1 mg/ml, Merck, Darmstadt, Germany).

### RNA extraction and quantitative real-time PCR (qRT-PCR)

Total RNA was extracted from transfected cells using TRIzol (Invitrogen) according to the manufacturer’s instructions and stored at − 80 °C. RSK4 mRNA in stably transfected clones was identified using reverse transcription-PCR and the SYBR Green II kit (Takara, Shiga-ken, Japan). The GAPDH gene was used as an internal control. The RSK4 primers used were 5`-TGAGTGGTGGAAACTGGGACAATA-3`(F) and 5`-TGGCATGGACTGTGGTCATGAGTC − 3`(R), and the primers used for GAPDH were 5`-GCACCGTCAAGGCTGAGAAC-3`(F) and 5`-TGGTGAA GACGCCAGTGGA-3`(R). The annealing temperature used was 60 °C.

### Western blot

Cells were lysed in buffer (50 mmol/l Tris-HCl, 150 nmol/l NaCl, 1 mmol/l EDTA, 1 mmol/l DTT, 0.1% Tween-20, 1 mmol/l phenylmethylsulfonyl fluoride, 10 mmol/l h-glycerophosphate, 1 mmol/l NaF, 2 mmol/l Na_3_VO_4_, 1–5 mg/ml leupeptin, and 1–5 mg/ml aprotinin). Protein was quantitated using a BCA protein assay (Pierce, Thermo). Protein aliquots (50 mg per lane) were separated using 10% SDS-PAGE, transferred onto a PVDF membrane (Millipore, Billerica, MA, USA), and visualised using chemiluminescence (Pierce, Thermo). Rabbit anti-human RSK4, matrix metalloproteinase 9 (MMP-9), CD44 (Sigma, USA), GSK-3β, phospho-GSK-3β (Ser9), β-catenin, rps6, and p-rps6 antibodies (Cell Signaling Technology, Inc., USA) were used for immunoblotting.

### Matrigel invasion assay

Cells were suspended at 5 × 10^5^ cells per ml in 1640 medium containing 1% FBS. 100 ml of the cell suspension was added to the upper well of Transwell inserts coated with 1mgml-1 Matrigel (Corning, Corning, NY, USA). Six hundred ml of 1640 medium with 20% FBS was added to the lower wells. The plates were incubated for 24 h at 37 °C in 5% CO_2_. After incubation, the inserts were carefully lifted and cells from the upper surface were gently scraped off. The remaining cells at the bottom of the filter were fixed and stained with 0.1% crystal violet. Each experiment was repeated at least three times to ensure reproducibility of the results.

### Statistical analysis

Chi-square tests were used to assess the difference and correlation between the expression of RSK4, CD44, and MMP-9 and the clinicopathological features of mRCC patients. Chi-square tests were also used to assess the correlation between the protein expression results and patient prognosis. All tests were performed using the Statistical Program for Social Sciences (SPSS) software (version 13.0, SPSS Inc., Chicago, IL, USA) two-sided. *P*-values< 0.05 were considered significant. The survival curves were constructed using a Kaplan–Meier analysis. The differences between curves were tested using the log-rank test.

## Results

### Expression of RSK4, CD44 and MMP-9 is unregulated in mRCC than in pRCC

**Fig. 1:**
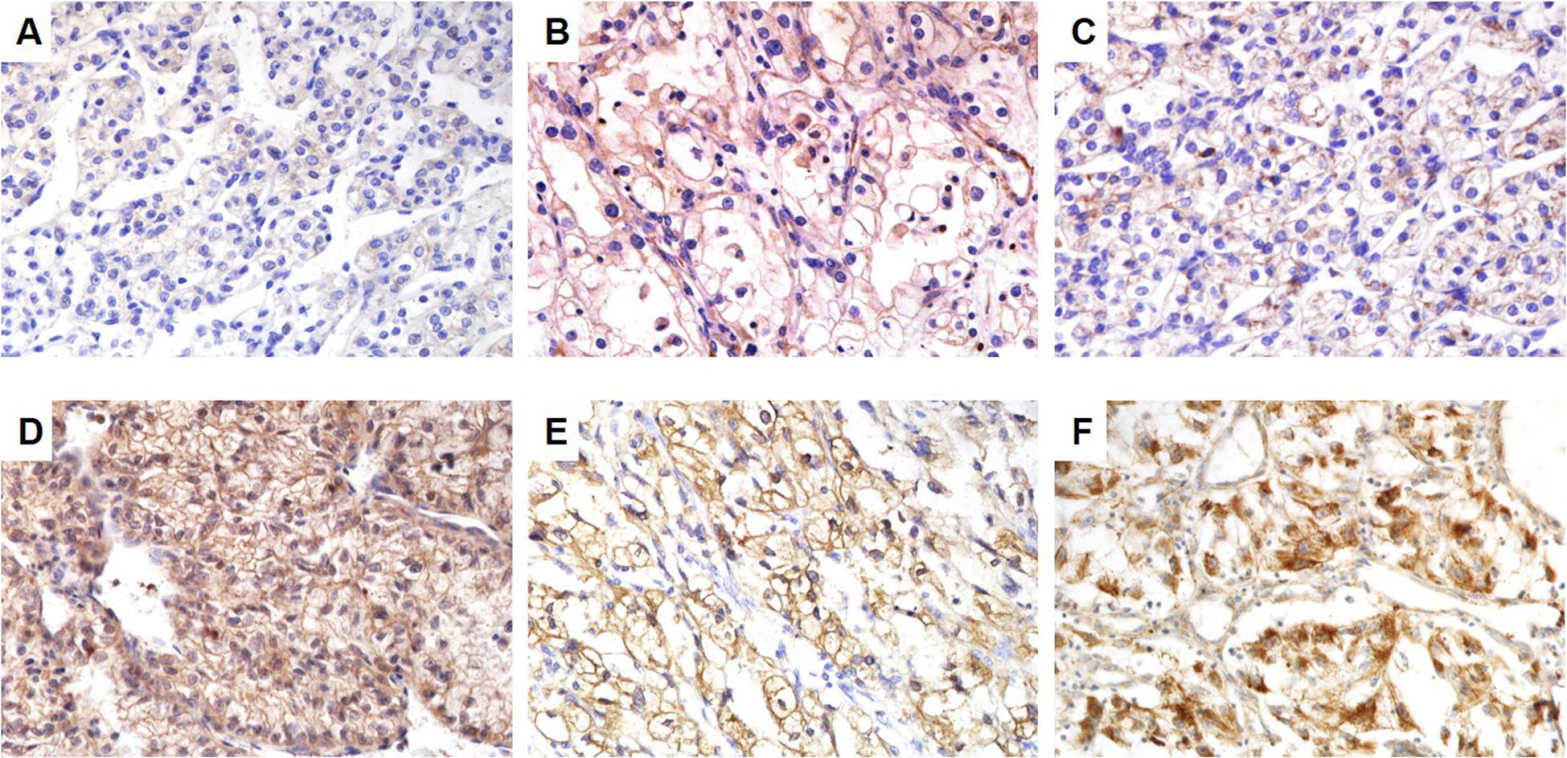
The expression of the RSK4, CD44 and MMP-9 proteins varies in primary ccRCCs and metastatic ccRCCs. **a**, **d**; **b**, **e**; **c**, **f** are form the same patients. **a**-**c**: primary ccRCCs, **d**-**e**: metastatic ccRCCs, × 400. **a** weak positivity for RSK4 protein. **b** weak positivity for CD44 protein. **c** weak positivity for MMP-9 protein. **d** positivity for RSK4 protein. **e** positivity for CD44 protein. **f** positivity for MMP-9 protein

The positive expression rates of RSK4, CD44 and MMP-9 in pRCC were 44.2% (Fig. 1a), 34.6% (Fig. 1b) and 69.2% (Fig. 1c), respectively. The positive expression rates of RSK4, CD44 and MMP-9 in pRCC were 75% (Fig. 1d), 68.75% (Fig. 1e) and 91.7% (Fig. 1f), respectively. Furthermore, in 48 mRCC samples, the expression of the three proteins was much higher than that in the 52 pRCC samples. A χ^2^ test showed that RSK4 was overexpressed in mRCCs compared with pRCCs (*P* = 0.002); similarly, a χ^2^ test showed that the difference in CD44 and MMP-9 expression in mRCC compared to pRCC was statistically significant (*P*_CD44_ = 0.001, *P*_MMP-9_ = 0.0002, Table 1). Moreover, statistical analysis found positive correlations among the RSK4, CD44 and MMP-9 expression levels (*P* = 0.008) in mRCC, and further analyses showed positive correlations between RSK4 and CD44 (*r* = 0.337, *P* = 0.019) and CD44 and MMP-9 (*r* = 0.285, *P* = 0.05). However, the correlation between RSK4 and MMP-9 was not significant (*r* = 0.000, *P* = 1.00, Table 2).

**Table 1:** χ^2^ test of the expression of RSK4, CD44 and MMP-9 in pRCCs and mRCCs

	RSK4		CD44		MMP-9	
	+	–	+	–	+	–
pRCC(*n* = 52)	23	29	18	34	36	16
mRCC(*n* = 48)	36	12	33	15	44	3
χ^2^	9.769		11.638		9.466	
*P*-value	**0.002**		**0.001**		**0.002**

**Table 2:** Correlations of the expression levels of RSK4, CD44 and MMP-9 in mRCCs

CD44	RSK4		Total	r	*P*-value
	+	–			
+	28	5	33	0.337	**0.019**
–	8	7	15		
Total	36	12	48		
CD44	MMP-9		Total	r	*P*-value
	+	–			
+	32	1	33	0.285	**0.050**
–	12	3	15		
Total	44	4	48		
MMP-9	RSK4		Total	r	*P*-value
	+	–			
+	33	11	44	0.000	1.000
–	3	1	4		
Total	36	12	48		

### The expression of the RSK4, CD44 and MMP-9 proteins is related to clinical features

On the other hand, the expression of RSK4, CD44 and MMP-9 was unrelated to age, gender, or metastatic sites (*P* > 0.05) but it was related to WHO/ISUP nucleolar grade (*P*_RSK4_ = 0.019; *P*_CD44_ = 0.026; *P*_MMP-9_ = 0.049, Table 3).

**Table 3:** RSK4, CD44 and MMP-9 immunostaining was cross tabulated with. Clinicopathological features of mRCC cases

	n	RSK4		CD44		MMP-9	
		+	–	+	–	+	–
Age		*P* = 0.094		*P* = 0.482		*P* = 0.382	
> 58	22 (45.8%)	14	8	14	8	21	1
≦58	26 (54.2%)	22	4	19	7	23	3
Gender		*P* = 0.098		*P* = 0.295		*P* = 0.388	
male	41 (85.4%)	29	12	27	14	37	4
female	7 (14.6%)	7	0	6	1	7	0
WHO/ISUP nucleolar grade		***P*** **= 0.019**		***P*** **= 0.026**		***P*** **= 0.049**	
1–2	33 (68.8%)	28	5	26	7	28	5
3–4	15 (31.2%)	8	7	7	8	8	7
Metastatic sites		*P* = 0.216		*P* = 0.458		*P* = 0.658	
bone	23 (23%)	15	8	16	7	21	2
lung	6 (6%)	6	0	3	3	5	1
brain	5 (5%)	5	0	3	2	4	1
soft tissue	10 (10%)	7	3	9	1	10	0
others	2 (2%)	2	0	1	1	2	0

### Overexpression of RSK4, CD44 and MMP-9 may predict poor outcome in mRCC patients

**Fig. 2:**
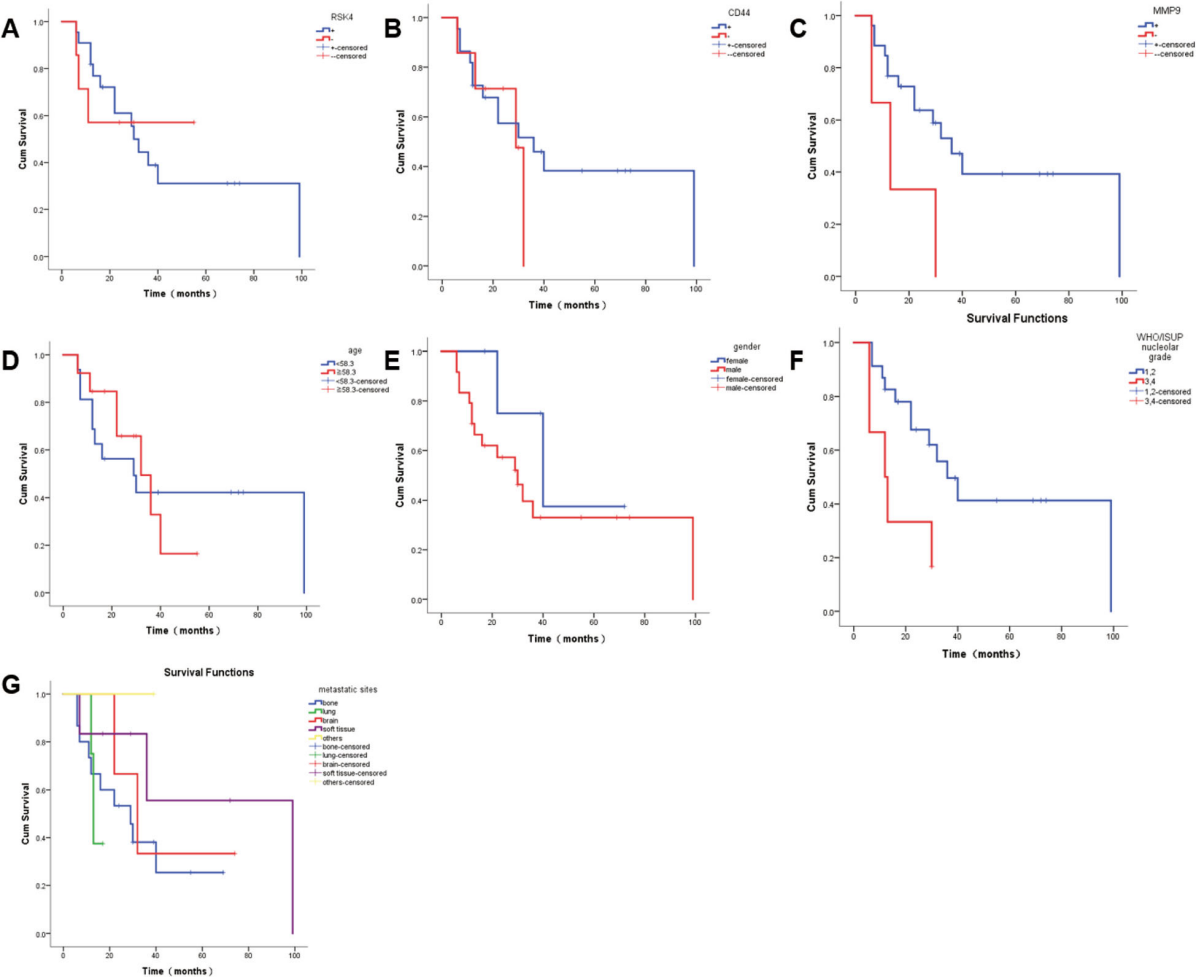
Positive expression of MMP-9 predicts poor outcome of metastatic ccRCCs patients. The Kaplan-Meier survival curve showed the association between MMP-9 positive and negative expression in 29 metastatic ccRCCs patients (*P* = 0.038), and similar association was observed for WHO/ISUP nucleolar grade 1–2 and 3–4 (*P* = 0.018). However, RSK4, CD44, age, gender and metastatic sites were not statistically significant (*P* > 0.05). Figure **a**-**f**, blue line represents positive while red line represents negative. In figure **g**, blue, green, red, purple, and yellow represent bone, lung, brain, soft tissues and other metastatic sites, respectively. (**a**) RSK4; (**b**) CD44; (**c**) MMP-9; (**d**) age; (**e**) gender; (**f**) WHO/ISUP nucleolar grade; (**g**) metastatic sites

Except for some censored cases, 29/48 patients were followed up for survival. Among them, 26 cases were MMP-9 positive. The median survival was 36 months in patients with MMP-9-positive tumours and 13 months in patients with MMP-9-negative tumours, which indicates that patients with MMP-9-positive tumours may experience tumour metastasis earlier. A log-rank test showed that the MMP-9-positive patients had a significantly worse survival than the MMP-9-negative patients (*P* = 0.038, Table 4, Fig. 2c). Similarly, WHO/ISUP nucleolar grade was associated with poor survival (*P* = 0.018, Table 4, Fig. 2f). The difference between the expression levels of RSK4 and CD44 among mRCC patients with different genders or ages was not statistically significant and neither were other clinicopathological features (*P* > 0.05, Table 4, Fig. 2a, b, d, e, g). Multivariate analysis revealed that MMP-9 expression (HR: 0.282, *P* = 0.050), WHO/ISUP nucleolar grade (HR: 0.286, *P* = 0.029) were independent prognostic indicators of survival (Table 4).

**Table 4:** Univariate and multivariate analysis of disease-specific survival in 29 mRCC cases with follow-up information

Variable	n	Mean survival in month (95% CI)	Univariate *P*-value	HR(95% CI)	Multivariate *P*-value
RSK4			0.959	0.333	0.409
+	22	30			
-	7	32			
CD44			0.581	0.949	0.959
+	22	36			
-	7	29			
MMP-9			**0.038**	0.282	**0.050**
+	26	36			
-	3	13			
age			0.329	0.482	1.623
≥ 55	9	13			
< 55	20	36			
gender			0.300	0.285	2.465
male	24	30			
female	5	40			
WHO/ISUP nucleolar grade			**0.018**	0.286	**0.029**
1~2	23	36			
3~4	6	12			
Metastatic sites			0.529	0.729	0.187
bone	15	24			
lung	4	12.5			
brain	3	32			
soft tissue	6	32.5			
others	1	39

### RSK4 enhances the invasive and metastatic ability of RCC cells by regulating the expression of MMP-9 and CD44

**Fig. 3:**
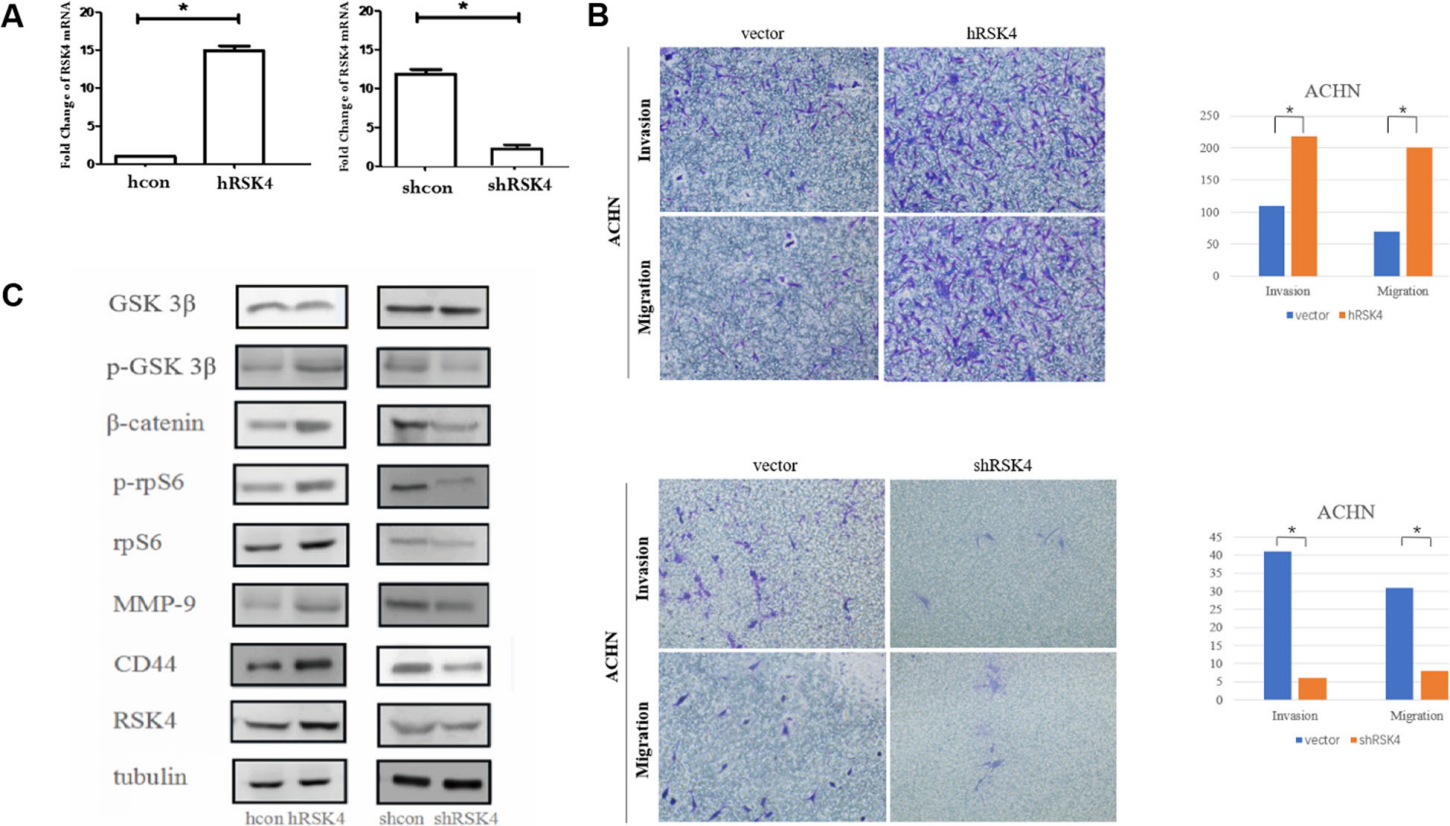
RSK4 promotes the invasive and metastatic activities of RCC cells. **a** qRT-PCR showed increased levels of RSK4 mRNA and protein (*P < 0.05). **b** RSK4-overexpressing ACHN cells showed elevated numbers of invasive and metastatic cells compared with the vector control cells (above, *P < 0.05 vs vector). When RSK4 was downregulated by shRNA, the number of invasive and metastatic cells was reduced (below, *P < 0.05 vs vector). **c** RSK4-overexpressing ACHN cells showed increased levels of CD44 and MMP-9; however, RSK4-suppressed cells had decreased levels of CD44 and MMP-9. Tubulin was used as a loading control. Moreover, RSK4-overexpressing ACHN cells showed increased levels of pGSK-3β^Ser9^, β-catenin, rps6 and p-rps6, whereas in the opposite was observed for the RSK4-suppressed cells

We generated an RSK4-overexpressing cell line by transfection with a pcDNA3.1/neo-RSK4 plasmid into the ACHN cell line. In addition, ACHN cells were transfected with shRSK4 lentivirus particles to downregulate the expression of RSK4. qRT-PCR and western blot analysis verified the changes in RSK4 expression in target cells. Compared with normal cells, RSK4 clones showed higher RSK4 mRNA and protein expression in RSK4-overexpressing ACHN cells and lower RSK4 expression in downregulated shRSK4 clones (Fig. 3a).

**Fig. 4:**
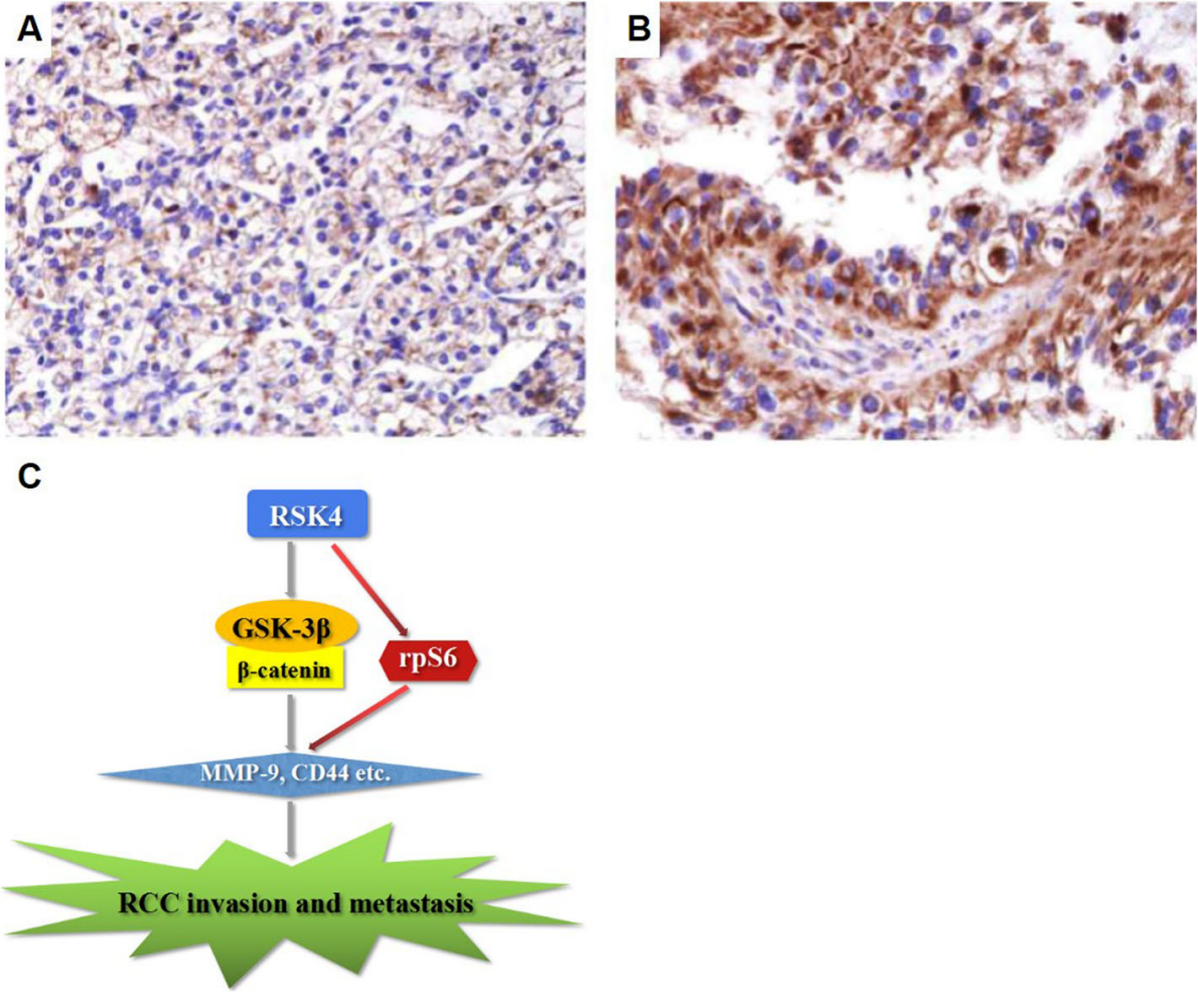
The expression of β-catenin protein varies in primary ccRCCs and metastatic ccRCCs. **a**: primary ccRCCs, **b**: metastatic ccRCCs, × 400. **a** weak positivity for β-catenin protein. **b** positivity for β-catenin protein. **c** Schematic of the pathway of RCC invasion and metastasis. RSK4 may be mediated through the regulation of CD44 and MMP-9 expression, while RSK4-mediated CD44 and MMP-9 may be mediated through the GSK 3β, β-catenin pathway or rps6 phosphorylation

The ability of RSK4 to affect the invasion and migration of RCC cells was evaluated in a Matrigel invasion assay. RSK4-overexpressing ACHN cells demonstrated significantly increased invasion and migration compared with the vector control cells (*P* < 0.05), but the cells infected with shRSK4 manifested a significant reduction in cell invasion and migration (*P* < 0.05, Fig. 3b). To further explore the mechanisms promoting invasion and migration, we evaluated the expression levels of CD44 and MMP-9, potential downstream factors of the RSK pathway. RSK4-overexpressing ACHN cells demonstrated increased CD44 and MMP-9 expression, and the opposite was observed in RSK4-knockdown cells (*P* < 0.05, Fig. 3c). These results suggest that the promotion of the invasion and migration of RCC cells by RSK4 could be mediated through the regulation of CD44 and MMP-9 expression. To determine how RSK4 regulates MMP-9 and CD44, we detected the levels of GSK 3β, pGSK-3β^Ser9^, β-catenin, rps6 and p-rps6, and the results suggested that RSK4-overexpressing ACHN cells demonstrated increased expression of pGSK-3β^Ser9^, β-catenin, rps6 and p-rps6. Silencing RSK4 expression decreased their expression (Fig. 3c). These results suggest that the promotion of the invasion and migration of RCC cells by RSK4 could be mediated through the regulation of CD44 and MMP-9 expression, which in turn may be mediated through the GSK 3β, β-catenin pathway or rps6 phosphorylation (Fig. 4c).

### The expression of β-catenin is higher in mRCC than pRCC tissues

The positive expression rate of β-catenin in pRCC was 39.1% (Fig. 4a), whereas the positive expression rate of β-catenin in mRCC was 65.7% (Fig. 4b). In mRCC samples, the expression of β-catenin was much higher than that in pRCC samples. A χ^2^ test showed that β-catenin was overexpressed in mRCCs compared with pRCCs (*P* = 0.046, Table 5). In summary, β-catenin may play an important role in RCC metastasis (Fig. 4c).

**Table 5:** χ^2^ test of the expression of β-catenin in pRCCs and mRCCs

	β-catenin	
	+	–
pRCC(*n* = 23)	9	14
mRCC(*n* = 35)	23	12
χ^2^	3.966	
*P*-value	**0.046**	

## Discussion

RCC, one of the most common malignant tumours in the urinary system, is difficult to diagnose in the early stages [1]. Researchers have comprehensively explored the pathogenesis and metastasis of RCC, evaluated the diagnostic and therapeutic methods, analysed molecules related to metastasis, and developed techniques for effective prognosis prediction [7, 8].

RSK4 is a member of the ribosomal S6 kinase (RSK) family, reported to be a downstream factor of the Ras-MEK-ERK signalling pathway, but its functions remain largely unknown [9]. There are only a few studies on the distribution of RSK4 mRNA in human normal tissues. In most studies, RSK4 is considered a potential tumour suppressing gene, and it has been reported that RSK4 expression is reduced in some tumours and RSK4 overexpression could inhibit the invasion and metastasis of tumour cells [4, 10]. In our previous study, the expression of RSK4 in RCC was higher than in normal kidneys, and the overexpression of RSK4 in RCC may lead to invasion and metastasis [5], suggesting that RSK4 may play a crucial role in RCC. In our study, we subsequently detected the expression of RSK4 by immunohistochemistry in a larger number of samples of primary RCC and metastatic RCC and found that RSK4 was overexpressed in mRCCs (*P* = 0.002).

MMP-9 plays an important role in the invasion and metastasis of tumours. The activation of the proto-oncogene may lead to the accumulation of MMP-9 transcripts and the production of MMP-9 protein [11]. The mature and activated MMP-9 could facilitate the invasion and metastasis of tumours through gelatine in the extracellular matrix [6]. The CD44 molecule on the surface of cancer cells interacts with molecules in endothelial cells, resulting in termination of cell circulation within the blood as the cell reaches the target organ. The variant protein of CD44 could alter the biological behaviour of cells and promote cell invasion and metastasis by affecting the skeletal and signal transmission systems in cells [12].

The interaction of CD44 and its ligand may induce cells to absorb and degrade hyaluronic acid, thereby enhancing cell migration [13]. The activated Ras−MEK − ERK signalling pathway can modulate the transcriptional expression of MMP-9 [14], which can interact with the CD44 extracellular domain. This interaction allows the secretion and activation of MMP-9, leading to the release of the CD44 intracellular domain (CD44ICD). Consequently, CD44ICD acts as a signal transduction molecule and translocates to the nucleus to mediate the transcription of CD44 itself [15]. MMP-9 can also enhance the contractibility of actin and p-MLC2 levels through CD44. This phenomenon increases the level of p-STAT3, resulting in the increased expression and secretion of MMP-9 and the formation of a positive feedback loop to maintain the invasion and metastasis of tumour cells [16]. We found that the expression of MMP-9 and CD44 was stronger in mRCC samples (*P* < 0.05), and further analysis showed that the expression of the three proteins was closely related (*P* = 0.008). In particular, the expression of RSK4 and CD44 (*P* = 0.019) and MMP-9 and CD44 (*P* = 0.05) were correlated.

GSK3β, a downstream mediator of the Wnt signalling pathway, can be regulated by RSK4. Binding of WNT to its receptor complex inhibits the degradation of β-catenin [17], thereby promoting tumourigenesis [18, 19]. rpS6 and the 70 kDa ribosomal protein S6 kinase (p70S6K), the downstream mediators of the mTOR signal pathway, can be phosphorylated at site Ser235/236 by RSK family members [20], while GSK3β, a downstream mediator of the Wnt signal pathway, can also be regulated by RSK4. Whether RSK4 could mediate the invasion and metastasis of renal cell carcinoma by rpS6 and GSK3β is also a new direction proposed by this study. Therefore, to preliminarily investigate the function and mechanism of the RSK4, CD44 and MMP-9 proteins in RCC, up- and downregulation of RSK4 were performed by lipofectamine-mediated gene transfection in ACHN cell lines, and the expression of CD44, MMP-9, rpS6, p-rpS6, GSK3β, pGSK-3β^Ser9^ and β-catenin levels in the RSK4-overexpressing ACHN cell line was studied by western blot. RSK4-overexpressing ACHN cells showed an increased expression level, whereas the opposite was observed in the RSK4-suppressed cells.

## Conclusions

In summary, we found for the first time that RSK4, MMP-9 and CD44 are overexpressed in metastatic ccRCC compared with primary ccRCC and that their expression is positively correlated. The high expression of MMP-9 was correlated with poor prognosis. In ccRCC, RSK4 may regulate MMP-9 and CD44 by the GSK 3β-β-catenin or rps6 phosphorylation pathway. This difference may indicate the potential mechanism of ccRCC tumour migration and invasion. However, the detailed underlying mechanism must be further investigated through molecular experiments.

## Supplementary information

**Additional file 1.**

**Additional file 2.**

**Additional file 3.**

## Data Availability

The data are available upon request on the following e-mail address: maj831@fmmu.edu.cn

## Abbreviations

RSK4: Ribosomal S6 protein kinase 4
pRCC: Primary renal cell carcinoma
mRCC: Metastatic renal cell carcinoma
TMA: Tumour organ tissue arrays
