# Supplementary figures and images for: Expression of RSK4, CD44 and MMP-9 is upregulated and positively correlated in metastatic ccRCC
Source: Diagn Pathol. 2020 Mar 24;15:28. doi: 10.1186/s13000-020-00948-6 (PMC7093975; doi:10.1186/s13000-020-00948-6)

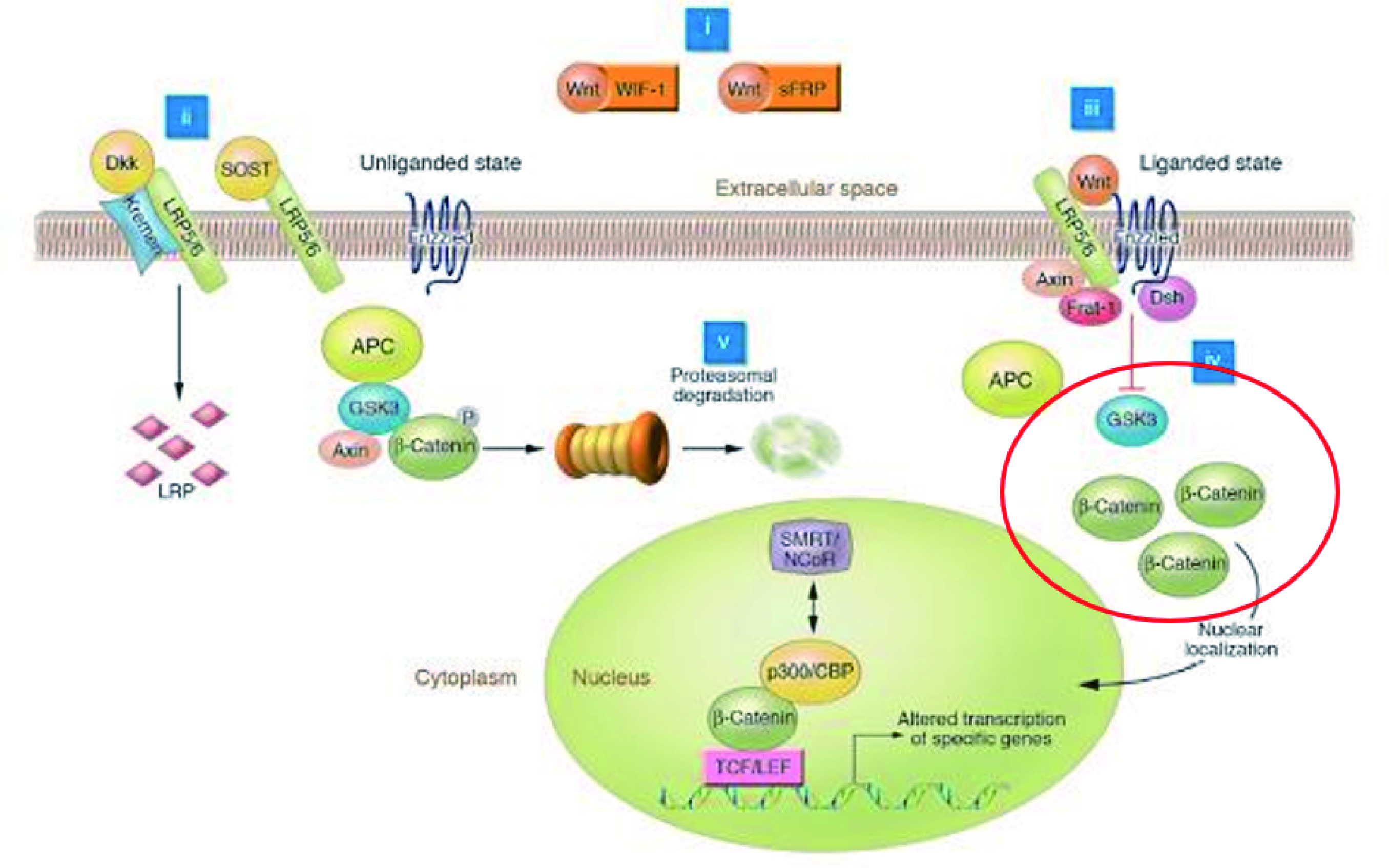

Supplement: Supplementary file 2 — Additional file 2. [file 13000_2020_948_MOESM2_ESM.tif]

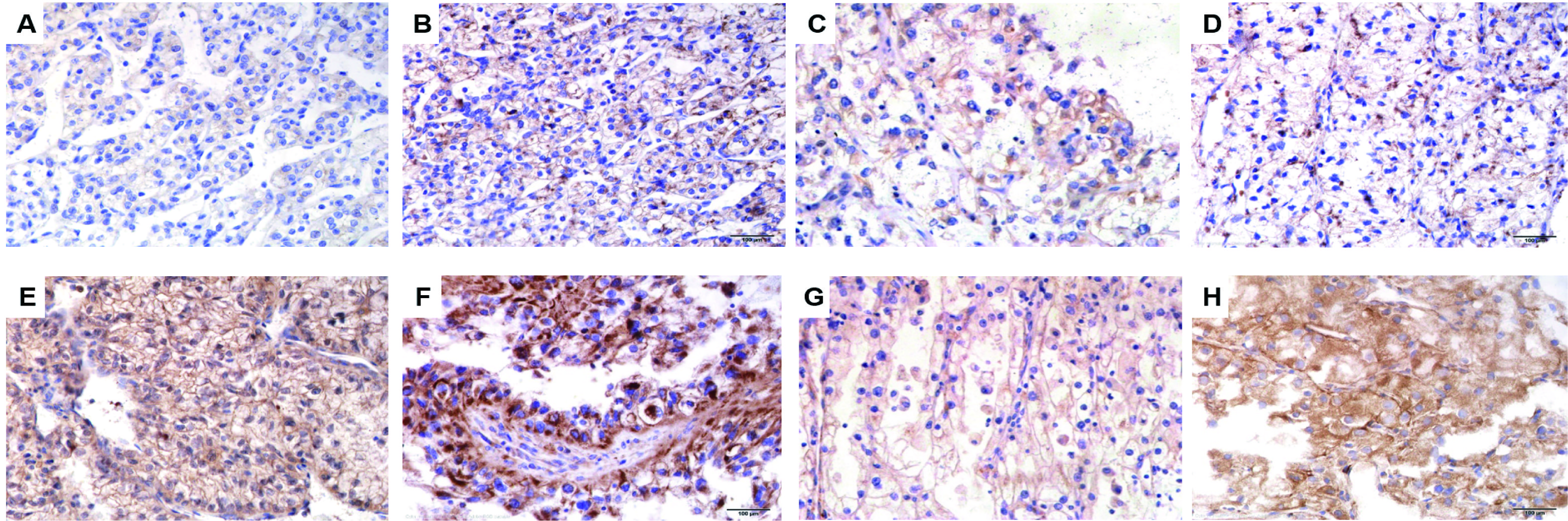

Supplement: Supplementary file 3 — Additional file 3. [file 13000_2020_948_MOESM3_ESM.tif]
